# Construct Validation of a Remote Brain Health Assessment Battery to Evaluate Vocational Aptitude and Factors Associated With Cognitive Resilience in the Military: Observational Trial

**DOI:** 10.2196/99490

**Published:** 2026-07-06

**Authors:** Mouna Attarha, Melissa Polusny, Melissa Fisher, Karrie Fitzpatrick, Wendy Schlinsog, Cathy Chen, Sophia Vinogradov

**Affiliations:** 1 Posit Science Corporation San Francisco, CA United States; 2 Department of Psychiatry & Behavioral Sciences University of Minnesota Medical School Minneapolis, MN United States

**Keywords:** military eligibility, vocational aptitude, cognitive resilience, ARMOR study, Advancing Research on Mechanisms of Resilience, National Guard, military recruits, brain health assessment, cognitive assessment, assessment scalability

## Abstract

**Background:**

Vocational aptitude and cognitive resilience predict military success, yet current assessments rely on resource-intensive, in-person testing that limits scalability. A brief, self-administered, remotely deployable computerized battery offers a practical solution for large-scale screening and monitoring.

**Objective:**

This study aims to deploy a set of computerized assessments among National Guard recruits and assess their preliminary construct validity against a standardized aptitude measure and a research-based proxy for cognitive resilience.

**Methods:**

In this observational study, 267 enlisted service members from the Minnesota Army National Guard participated in 2 complementary ethics-approved observational trials: Office of Naval Research Neuropsychometrics and Advancing Research on Mechanisms of Resilience (ARMOR). National Guard soldiers in ARMOR completed the Armed Forces Qualification Test (AFQT), Penn Computerized Neurocognitive Battery (Penn CNB), and a 20-minute computerized brain health assessment battery (BrainHQ) at separate time points over the course of their military careers. BrainHQ assessments consisted of adaptive psychophysical tasks measuring the speed and accuracy of visual and auditory information processing. The battery assessed decision-making speed, emotion-processing speed, selective attention under speeded conditions, working memory capacity for speeded visual elements, verbal memory and learning of speeded speech, and problem-solving speed. The Penn CNB included nonspeeded neuropsychological assessments of executive function, verbal memory, social cognition, and reasoning. Linear regression evaluated the association between BrainHQ performance and AFQT percentiles, and partial correlations assessed associations between conceptually related BrainHQ and Penn CNB subtests.

**Results:**

Participants were predominantly young (mean age 19.1 years) and male (178/267, 66.7%). BrainHQ performance was significantly associated with enlistment eligibility and vocational aptitude, as measured by the AFQT (*P*<.001), after controlling for age and education. The overall model explained 24.4% of the variance in AFQT percentiles (adjusted R2=0.227). The BrainHQ assessment composite was the strongest predictor, uniquely accounting for 19.2% of the variance and supporting the construct validity of aptitude. These associations persisted despite the temporal separation between assessment time points. Quartile analyses showed graded relationships between BrainHQ performance and AFQT eligibility thresholds, with higher BrainHQ performance associated with progressively greater probabilities of meeting higher AFQT benchmarks. Preplanned partial correlations between BrainHQ subtests and standardized neurocognitive measures from the Penn CNB showed significant positive associations (r=0.17-0.25; all *P*<.001 to .02) with cognitive domains typically associated with cognitive resilience.

**Conclusions:**

A brief, self-administered, and scalable brain health battery demonstrates associations with military vocational aptitude and with neurocognitive domains associated with cognitive resilience. Future studies should evaluate whether integrating these assessments into current practices predicts success in Basic Combat Training, guides military progression, and supports long-term cognitive screening and monitoring across the Armed Forces.

## Introduction

### Background

Modern military operations require scalable, efficient tools to assess vocational aptitude and resilience to sustain operational effectiveness over time [1]. Within military science, vocational aptitude typically refers to foundational cognitive abilities and general content knowledge that support the acquisition, integration, and execution of mission-relevant operations [2], influencing training success, occupational placement, and subsequent job performance [3].

The concept of cognitive resilience remains actively debated across disciplines, with definitions varying across psychological, neurological, and military contexts [4,5]. In many frameworks, resilience is used broadly to include emotional, physical, and psychosocial constructs; however, such definitions can obscure measurement specificity in cognitive research. Consensus definitions from the National Institutes of Health (NIH)–funded Collaboratory on Research Definitions for Reserve and Resilience in Cognitive Aging and Dementia [6] describe cognitive resilience as a dynamic process shaped by interacting protective factors, including cognitive reserve, brain reserve, and brain maintenance. Cognitive reserve reflects adaptive neural processing that enables individuals to perform better than expected given neural challenges, while brain reserve reflects neurobiological and structural endowment, such as neuromodulatory control or neuronal and synaptic densities, that provides a buffer against decline. Brain maintenance emphasizes the relative preservation of neural integrity over time, with slower accumulation of age- or disease-related pathology supporting sustained cognitive performance. Although developed in aging research, these constructs align conceptually with military priorities focused on sustaining cognitive abilities to perform mission-relevant duties reliably and safely under conditions of stress, fatigue, or evolving operational demands [4,7].

### Motivation

Evaluations of aptitude and resilience may help identify vulnerabilities early to enable targeted interventions that reduce attrition, protect health, and support success [8,9]. These assessments have historically integrated medical fitness, training proficiency, psychological health, and cognitive performance. Large-scale screening tools such as the Armed Services Vocational Aptitude Battery (ASVAB) and its derived Armed Forces Qualification Test (AFQT) have been used to assess military eligibility relevant to vocational placement and have demonstrated predictive associations with training completion, occupational proficiency, and retention, thereby providing an important benchmark for aptitude within military populations [2,10-12].

In parallel, research-focused neurocognitive batteries, including the Penn Computerized Neurocognitive Battery (Penn CNB), have been used to evaluate functions associated with adaptive cognitive functioning [13]. While the Penn CNB was not designed as a measure of cognitive resilience, it has been used in prior studies to examine cognitive performance variability associated with stress exposure, psychiatric risk, and functional adaptation, making it a useful research-based proxy for cognitive domains implicated in resilience [14-17].

In addition to the ASVAB and Penn CNB, the Automated Neuropsychological Assessment Metrics [18] is a widely validated, standardized assessment that enables longitudinal monitoring of cognition, risk factors, and resilience-related outcomes across large military populations. While highly valuable, this and other established batteries often require supervised administration, specialized infrastructure, or optimization for specific use cases, such as concussion monitoring or suicidality, rather than scalable screening, profiling, or brain health monitoring. Recent advances in portable neural sensing technologies, such as functional near-infrared spectroscopy and electroencephalography, as well as emerging wearable devices integrated into headbands, earbuds, or helmets, offer promising complementary approaches for assessing cognitive states in real-world contexts [19-22]. These systems can capture real-time neural and physiological indicators of workload, fatigue, and attention during task performance, thereby providing ecologically valid data on cognitive resilience under operational stress. However, widespread implementation remains limited by practical challenges, including calibration, artifact and signal processing, interpretation, technical expertise, and constraints related to comfort, cost, and security [23]. These forms of evaluation are generally resource-intensive, time-consuming, and challenging to deploy across geographically dispersed and operationally diverse military forces.

There consequently remains a need for brief, self-administered, and remotely deployable computerized cognitive assessment batteries that can complement existing evaluation approaches by addressing critical gaps in efficiency and scalability. Here, we align with the mission of the Brain Health Initiative of the Department of Defense to develop neurologically informed assessments capable of evaluating core cognitive functions associated with military performance, consistent with Line of Effort 5 (LOE5) [24].

### Study Objective

Our objective was to evaluate whether performance on BrainHQ assessments demonstrates expected relationships with cognitive benchmarks collected in military research settings. We analyzed data from enlisted service members in the Minnesota Army National Guard participating in 2 complementary observational trials: the Office of Naval Research (ONR) Neuropsychometrics Study, designed to develop and implement a comprehensive brain health assessment battery to inform career progression and readiness for duty, and the Advancing Research on Mechanisms of Resilience (ARMOR) study, a prospective longitudinal investigation designed to identify neural, cognitive, behavioral, and social factors that promote resilience in the context of military stressors [25]. The goals of these parent studies were broader than those of this analysis.

This study focuses specifically on initial construct validation, with aptitude representing an individual’s cognitive potential to succeed in specific military occupational specialties and cognitive resilience representing the capacity to adaptively maintain stable cognitive performance when exposed to operational stressors such as those encountered during Basic Combat Training (BCT). National Guard soldiers completed the AFQT, Penn CNB, and BrainHQ assessments at separate time points over the course of their military careers (before or after BCT). Our objectives were 2-fold. The first was to establish the construct validity of BrainHQ composite scores against AFQT percentiles as an index of operational cognitive capability and vocational aptitude. The second was to establish the construct validity of BrainHQ measures against Penn CNB measures as an index of neurocognitive functions associated with cognitive resilience. We hypothesized that BrainHQ assessment performance would show positive associations with both the AFQT and Penn CNB measures, consistent with convergent validity for a scalable computerized battery suitable for military populations.

## Methods

### Ethical Considerations

The Neuropsychometrics study was reviewed and approved by the Institutional Review Board of the University of Minnesota (approval number STUDY00005620) and the Department of the Navy’s Human Research Protection Official (approval number MOD00032864). ARMOR study procedures, including recruitment for this study, were reviewed and approved by the institutional review boards of the University of Minnesota (approval number STUDY00004470) and the Minneapolis VA Health Care System (approval number VAM-18-00334/1594664) [17]. The study was conducted in accordance with applicable federal regulations and the principles outlined in the Declaration of Helsinki.

All participants provided informed consent before participation. Participants received written information describing the study purpose, procedures, requirements, potential risks and benefits, confidentiality protections, and the voluntary nature of participation, including that consent could be withdrawn at any time without penalty. A waiver of the requirement to document consent was granted for the survey component of the ARMOR study. ARMOR participants indicated their consent by entering a study ID number [17]. Consent forms for ONR Neuropsychometrics included an “I Consent” response option provided through the COMPAS data management system, along with completion of Unsecure Email and Unsecure Text Authorization forms as part of the consent process. Participants further consented to the use of their ARMOR study data and military records for the purposes of this study and the analyses reported herein (W Schlinsog et al, unpublished data, 2026). Completed consent forms for ONR Neuropsychometrics were saved to participants’ records in COMPAS and made available for download by participants. Participants received a US $40 Amazon electronic gift card as compensation for completing study activities. Compensation was not contingent on study performance.

Participant privacy and data confidentiality were protected through the use of deidentified study data and secure data storage procedures. Penn CNB assessment measures collected through ARMOR were stored on secure University of Minnesota servers. Demographic information and AFQT percentile scores were maintained on secure Minneapolis VA Health Care System servers and linked to Penn CNB scores and BrainHQ assessment data using study identification numbers. BrainHQ assessment data were collected and stored using deidentified usernames on the BrainHQ platform (W Schlinsog et al, unpublished data, 2026), which complies with the Health Insurance Portability and Accountability Act (HIPAA), System and Organization Controls 2 Type II, and National Institute of Standards and Technology Special Publication 800-171 security standards. Study data were restricted to authorized study personnel, and personally identifying information was removed before data integration. The analyses reported herein were conducted using deidentified datasets.

### Study Design and Participants

This observational study was conducted among enlisted soldiers in the Minnesota Army National Guard who were concurrently enrolled in ARMOR. ARMOR participants aged 17 years or older were enrolled between April 14, 2019, and October 16, 2021, with an assigned ship date for BCT. As defined by the ARMOR protocol, exclusion criteria included prior military service or any history of BCT participation [17]. Exclusion criteria in the ONR protocol included psychiatric conditions (eg, schizophrenia) or medical conditions (eg, traumatic brain injury) known to impair cognition in the opinion of the principal investigator (SV). Participants enrolled in the ARMOR study were recruited by the ARMOR study team to participate in the ONR study. Penn CNB measures were self-administered and completed at a single time point before shipping for BCT. BrainHQ measures were administered after BCT. Study details are available in the ARMOR [17] and ONR Neuropsychometrics protocols (W Schlinsog et al, unpublished data, 2026).

### Data Collection Procedures

ARMOR participants completed Penn CNB assessments independently on Chromebooks (Google LLC) in classroom settings at regional armories during predeployment as newly enlisted service members before shipping to basic training, following ARMOR protocol procedures. Approximately 18-24 months after returning from basic training, a subgroup of ARMOR participants received a recruitment email or SMS text message from ARMOR study staff regarding their interest in learning more about the ONR Neuropsychometrics study. The recruitment email contained a link to the University of Minnesota’s HIPAA-compliant Qualtrics platform with a single survey question asking whether ARMOR participants wanted to learn more about the ONR study using a yes/no response and, if so, requesting permission to share their contact information with the ONR study team. Interested participants received written instructions via email or SMS text message from ONR staff. The instructions included a clickable link to log in to the COMPAS data management system using a uniquely generated participant number to review study procedures, risks and benefits, and provide informed consent. After providing consent, participants remotely completed the ONR Neuropsychometrics study activities, including BrainHQ. Participants accessed BrainHQ using single sign-on and clicked a gray arrow on the page titled “Cognitive Check-In” to self-administer the 7 BrainHQ assessments (W Schlinsog et al, unpublished data, 2026). All assessments included a written tutorial with practice trials that participants completed on their own personal computers after BCT. ARMOR data included demographic information and AFQT percentiles extracted from military records obtained from the Minnesota National Guard. The ASVAB, from which AFQT scores were derived, was completed at a Military Entrance Processing Station for enlistment purposes and was not readministered as part of research participation in this study.

### Measures

#### Overview

The current investigation includes demographic and performance data from the AFQT, Penn CNB, and BrainHQ brain health assessments.

#### Demographic Information

Participant demographic data included age (years), biological sex (male or female), education based on the highest degree obtained (some high school, General Educational Development [GED]/high school diploma, some college/associate degree, 4-year college degree [BA or BS]/master’s degree [MA, MS, MBA, MPH, etc]/doctoral degree [PhD, MD, DD, etc]), race (White, Black or African American, American Indian or Alaska Native, Asian, Other, Native Hawaiian or Other Pacific Islander, multiracial), and ethnicity (Hispanic, not of Hispanic origin).

#### Armed Forces Qualification Test

The AFQT is used by the United States military to evaluate eligibility and align military personnel with roles that match their cognitive capabilities. Scores are derived from 4 subtests of the ASVAB: Arithmetic Reasoning, Word Knowledge, Paragraph Comprehension, and Mathematics Knowledge. Scores reflect percentile rank relative to a nationally representative sample of peers and are categorized into 5 eligibility bands: individuals qualifying for any military occupational specialties (93%-99%), high eligibility across most roles (65%-92%), general eligibility across roles (50%-64%), eligibility with some restrictions (31%-49%), and typically ineligible for enlistment (<30%).

#### Penn Computerized Neurocognitive Battery

##### The Penn CNB Battery

The Penn CNB is a validated computerized assessment battery [15] designed to measure key neurocognitive functions proposed to influence cognitive resilience in military research contexts [17,26]. As part of the ARMOR study, National Guard recruits completed the 4 Penn CNB subtests described below. The battery required approximately 20 minutes to complete.

##### Penn Continuous Performance Test

This test measures sustained attention and vigilance. Participants are presented with a series of stimuli and must respond only to target sequences, requiring continuous monitoring and inhibition of impulsive responses. Accurate and rapid target detection among distractors reflects an individual’s capacity to maintain focus, a key component of cognitive resilience under operational stress.

##### Penn Word Memory Test

This test measures episodic memory through auditory verbal learning and recall. Participants remember a list of words and later identify previously presented items among distractors. This task simulates scenarios in which accurate memory for verbal information is critical, such as recalling orders or locations. Strong performance reflects an individual’s ability to retain and retrieve mission-relevant information.

##### Penn Measured Emotion Differentiation Test

This test measures social cognition through the recognition and differentiation of subtle emotional expressions. Participants view progressively morphed images of human faces and identify the intensity of the depicted emotions. Emotion differentiation mirrors real-world interpersonal demands encountered in military settings, such as interpreting emotional cues during negotiation, deescalation, and team coordination.

##### Penn Verbal Reasoning Test

This test measures higher-order verbal reasoning and complex problem-solving. Participants are presented with pairs of word-based analogies and must determine the correct relationship. Flexible thinking and the ability to rapidly restructure understanding are associated with cognitive resilience.

#### BrainHQ Brain Health Assessments

##### BrainHQ Assessment Battery

**Figure 1:**
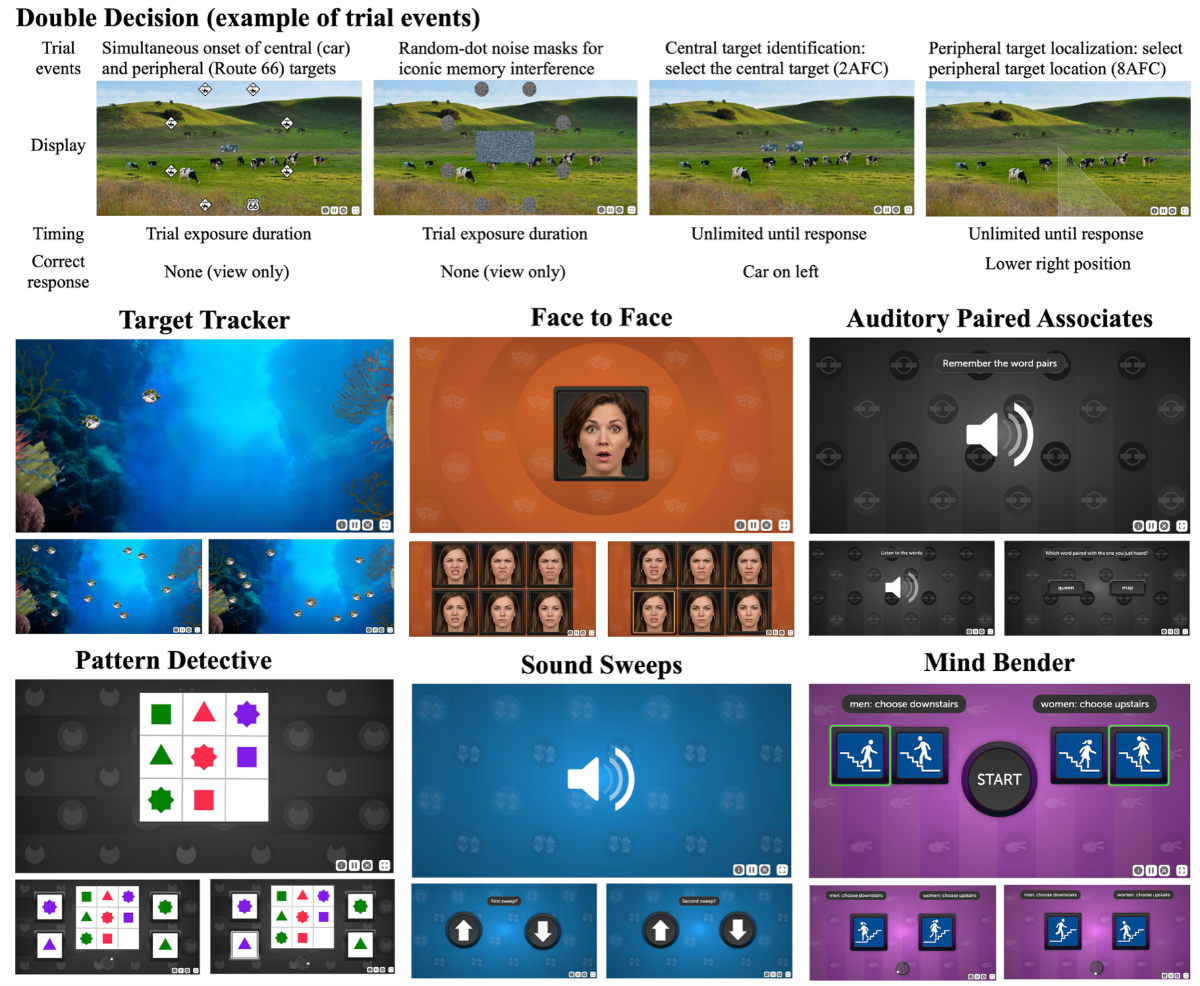
Sequence of trial events for the Double Decision computerized cognitive assessment. In this dual-task paradigm, participants simultaneously viewed a vehicle at the center of the screen and signs in the periphery presented for a brief exposure duration. Participants identified which of 2 vehicles was presented and the location at which the Route 66 sign appeared among 8 possible peripheral locations. Stimulus displays and response screens for the remaining assessments are presented in subsequent figures. All figures are reproduced, with permission, from Posit Science Corporation. The facial stimuli were generated using Generative AI (Gemini).

The brain health assessments were developed by Posit Science following neuroscientific principles described previously [27,28] (see Figure 1). Brain reserve plays a central role in the causal pathways connecting cognitive performance to operational outcomes [1,24,29]. BrainHQ brain health assessments use psychophysical procedures centered on the speed and accuracy of information processing (eg, spatiotemporal sampling rates and temporal integration windows) that influence discrimination speed, neuromodulatory network health, and the representational fidelity supporting the processing efficiency of memory, attention, and executive functioning [30]. Prior research has shown significant associations between BrainHQ and biological, cognitive, emotional, and functional measures, including physical health markers such as hypertension and atrial fibrillation [31]; brain imaging measures of neuromodulatory network integrity [28]; functional connectivity [32]; neural timing speed measured by electroencephalography [33,34]; standard neuropsychological batteries of cognition, including NIH EXAMINER, Montreal Cognitive Assessment, Mini-Mental State Examination, Stroop, Trail Making Tests A and B, Digit Symbol, and Benton Visual Retention [28,35,36]; coping strategies (W Schlinsog et al, unpublished data, 2026); and functional performance on activities of daily living, such as driving, and academic grade point averages (W Schlinsog et al, unpublished data, 2026) [37,38].

Subtests were selected based on their hypothesized relevance to military service under the premise that service members must efficiently process and respond to rapidly presented information while maintaining accuracy in complex, dynamic operational environments. Assessments targeted decision-making speed, problem-solving speed, auditory and visual memory capacity for speeded information, emotion-processing speed, and auditory and visual perceptual processing speed [27]. The battery took approximately 20 minutes to complete, and the subtests are described below [27].

##### Mind Bender

This assessment measures decision-making speed. In a task-switching paradigm, participants make decisions about competing stimuli based on rules that change from moment to moment. The adaptive dimension is display exposure duration, and scores are recorded in milliseconds, with lower scores indicating better performance (range 64-94,190 ms). The assessment targets the ability to operate effectively under shifting rules and rapidly changing circumstances.

##### Auditory Paired Associates

This assessment measures verbal memory and learning. Using an associative memory paradigm, participants recall the word associated with a cue presented during the learning phase. The adaptive dimension is set size, and scores are recorded as the number of word-pair associations recalled, with higher scores indicating better performance (range 1-12 pairs). The assessment targets the ability to link and accurately recall critical verbal information, such as commands, codes, or communication sequences.

##### Face to Face

This assessment measures the participant’s social and emotional processing. In this task, participants select the face with the same emotional expression as a previously presented target face. Facial expressions were produced by paid actors who consented to the publication and commercial use of their images. The adaptive dimension is display exposure duration, and scores are recorded in milliseconds, with lower scores indicating better performance (range 32-5623 ms). The assessment targets the ability to accurately discriminate subtle emotional expressions needed for targeting and threat assessment.

##### Pattern Detective

This assessment measures problem-solving skills. Using a visual matrix reasoning paradigm, participants are presented with an incomplete pattern and must choose the correct image from 4 available options within a speeded response window. The adaptive dimension is pattern complexity, with higher scores indicating better performance (range 1-8 complexity levels). The assessment targets the ability to identify patterns in complex visual environments under time pressure.

##### Target Tracker

This assessment measures divided attention and working memory capacity. In a multiple-object tracking paradigm, participants track a set of targets (defined by their spatiotemporal onset) among visually identical distractors. The adaptive dimension is set size, and scores are recorded as the number of objects tracked, with higher scores indicating better performance (range 1-10 items). The assessment targets the ability to attend to and remember multiple pieces of information simultaneously, supporting strategic planning and multitasking under pressure.

##### Double Decision

This assessment measures selective attention and visual processing speed. In this dual-task paradigm, participants discriminate between and identify which of 2 perceptually similar cars appeared in the center of gaze while simultaneously locating a traffic sign in the peripheral visual field. The adaptive dimension is display exposure duration, and scores are recorded in milliseconds, with lower scores indicating better performance (range 32-3162 ms). The assessment targets the speed with which recruits identify targets in their peripheral vision while maintaining focus on a central task, mimicking real-world operational scenarios.

##### Sound Sweeps

This assessment measures auditory processing speed. In a time-order judgment paradigm, 2 successive frequency-modulated tone sweeps are presented, and participants indicate whether the frequency increased or decreased within each tone. The adaptive dimension is sweep speed, and scores are recorded in milliseconds, with lower scores indicating better performance (range 16-1000 ms). The assessment targets the ability to rapidly interpret and respond to verbal instructions, environmental sounds, or radio communications in real time to support situational awareness.

The BrainHQ assessments use procedures that continuously adjust task difficulty to maintain participant performance at approximately 80% accuracy. When accuracy exceeds the criterion, subsequent trials become more difficult by reducing stimulus exposure duration (or increasing set size). Conversely, when accuracy falls below the criterion, task difficulty is reduced by increasing exposure duration (or decreasing set size). As a result, accuracy is incorporated directly into the scoring algorithm used to estimate performance thresholds, and the primary performance metrics reflect the minimum exposure duration (or maximum set size) that a participant can reliably maintain.

### Statistical Analyses

Demographic data were described using arithmetic means, SDs, and ranges for continuous variables (age, Penn CNB subtests, and BrainHQ subtests). For education, sex, race, and ethnicity, the number of participants selecting each response option was divided by the total number of respondents. Both raw counts and percentages for education, sex, race, and ethnicity are reported.

To evaluate associations between the BrainHQ assessments and military eligibility and vocational aptitude, ordinary least squares regression was used with AFQT percentile scores as the dependent variable and BrainHQ composite scores as the predictor. To derive the BrainHQ composite, the raw threshold scores (for Target Tracker, Pattern Detective, and Auditory Paired Associates) and the log-transformed raw millisecond threshold scores (for Double Decision, Sound Sweeps, Face to Face, and Mind Bender) were converted to cohort *z* scores for each subtest to place performance on the same standardized scale, where higher scores indicate better performance. The *z* scores were then averaged across the 7 BrainHQ subtests to generate the composite. Composite construction was aligned with the study protocol (W Schlinsog et al, unpublished data, 2026) and prior published studies using BrainHQ measures [27]. Age and education were included as covariates in the model because of their well-established influence on AFQT performance. Age was coded in years, whereas education was coded by the National Guard on a 1-4 scale (1=some high school, 2=GED or high school diploma, 3=some college or associate degree, and 4=bachelor’s degree or higher). The model handled missing data using listwise deletion. To assess potential selection bias due to missing data, we compared participants included in the regression model with those excluded because of missing AFQT, age, sex, ethnicity, or education data. Group comparisons were conducted using 2-tailed independent samples *t* tests for continuous variables and chi-square tests for categorical demographic variables.

To evaluate cognitive resilience, we used partial correlations between BrainHQ subtest scores and the primary performance metric for each Penn CNB subtest, controlling for age and education. Subtest comparisons were planned a priori based on the targeted cognitive domain. Mind Bender (*z* score) was correlated with the Penn Continuous Performance Test (CPT; d′) because both evaluate executive function. Face to Face (*z* score) was correlated with the Penn Measured Emotion Differentiation Test (36 items; MEDF36) (total correct responses) to assess social cognition. Pattern Detective (*z* score) was correlated with the Penn Verbal Reasoning Test (PVRT; total correct responses) to assess logical reasoning, and Auditory Paired Associates (*z* score) was correlated with the Penn Word Memory Test (CPW; d′) to assess word memory.

To further contextualize performance in operationally meaningful terms, we also present the relationship between BrainHQ composite scores and AFQT percentiles as the probability of exceeding specific AFQT benchmarks. Participants who completed the BrainHQ assessments were divided into 4 quartiles based on composite *z* score performance, with 1 representing the lowest-performing quartile and 4 representing the highest-performing quartile. For each quartile, we calculated the probability of participants meeting or exceeding established AFQT eligibility bands, reflecting cognitive aptitude for various military occupations, specifically ≥10 (representing those typically ineligible for enlistment), ≥31 (eligible with some restrictions), ≥50 (general eligibility across roles), ≥65 (high eligibility across most roles), and ≥93 (individuals qualifying for any military occupational specialty). For all analyses, *P*<.05 was considered statistically significant.

## Results

The ONR Neuropsychometrics study enrolled ARMOR participants between August 2022 and July 2025. All data were retrieved in July 2025.

Of 1201 eligible service members, 344 were recruited, and 269 completed study-related activities. The attrition rate was around 22% (77/344, 22.4%), reflecting ARMOR participants who initially expressed interest in participating in the ONR Neuropsychometrics study but did not subsequently complete ONR study activities. A total of 2 participants were excluded because of psychiatric or medical conditions. All participants with available data were included in the analyses that follow.

National Guard recruits (N=267) had a mean age of 19.13 years (SD 3.27 years; range 17-36 years). Participants were predominantly male (178/267, 66.7%), White (164/267, 61.4%), and had completed some high school education (93/267, 34.8%; see Table 1). A full characterization of the participant demographics and the CONSORT flow diagram are presented elsewhere (W Schlinsog et al, unpublished data, 2026).

**Figure 2:**
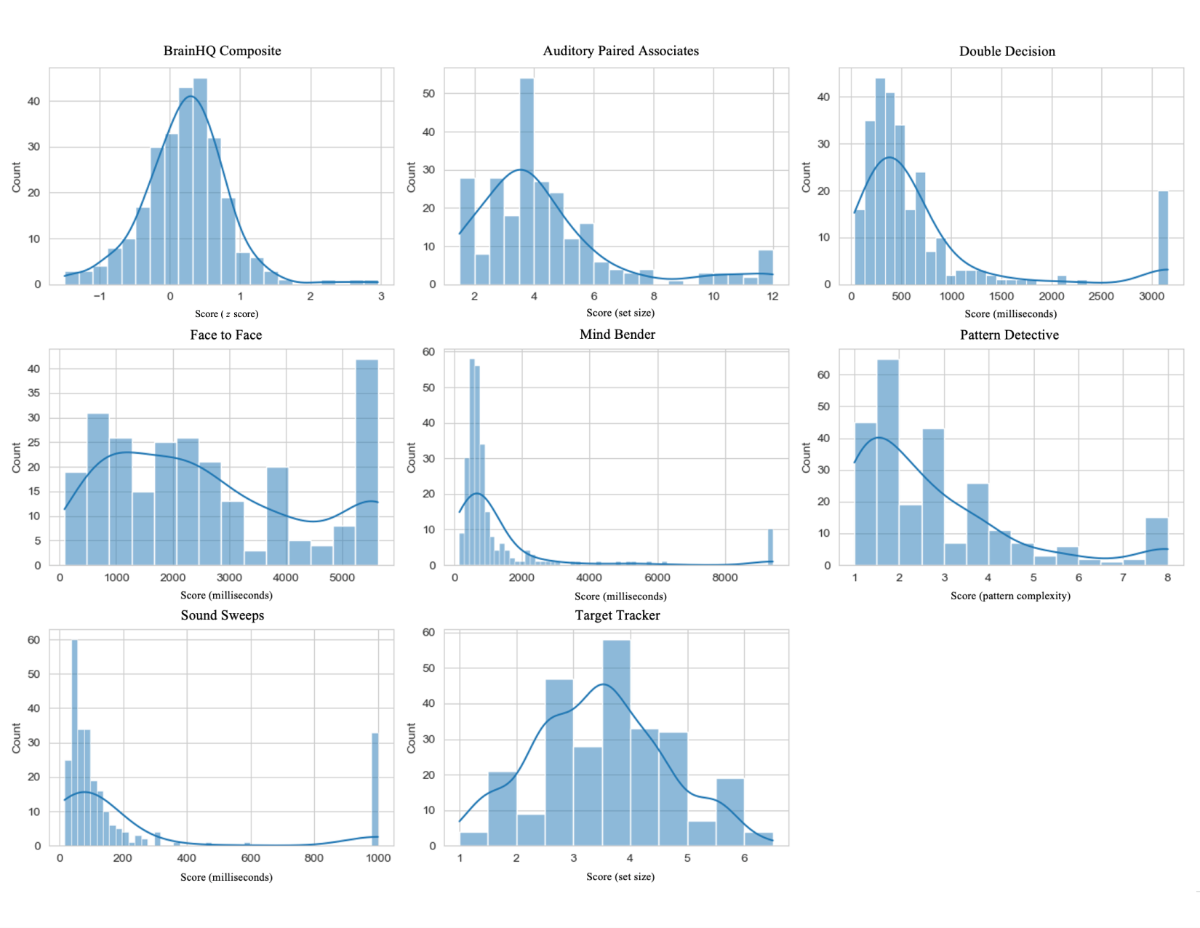
Histograms of performance on the BrainHQ composite (<i>z</i> score) and each BrainHQ subtest.

The mean AFQT percentile was 70.45 (SD 19.54; range 20-99). Descriptive statistics for the Penn CNB and BrainHQ subtests are provided in Table 1. BrainHQ performance histograms are shown in Figure 2.

The overall regression model, controlling for age and education, was statistically significant (*F*_5,225_=14.53, *P*<.001) and explained 24.4% of the variance in AFQT percentile scores (adjusted *R*^2^=0.227). Tests of model assumptions showed that the residuals were approximately homoscedastic (White test=19.32, *P*=.15) and not autocorrelated (Durbin-Watson statistic=1.95). Skewness (–0.43) and kurtosis (2.68) were within acceptable limits. The residuals were approximately normally distributed.

Individual predictors indicated that higher BrainHQ composite performance was significantly associated with higher AFQT scores (β=15.40, SE 2.10, t_255_=7.32, 95% CI 11.26-19.55, *P*<.001). The BrainHQ composite was the strongest predictor in the model, uniquely accounting for 19.2% of the variance (*sr*^2^=0.192), whereas the remaining variance was shared with, or explained by, the other predictors (education and age). Among the education categories, participants with some college degree showed a significant positive association with AFQT scores compared with those with some high school (β=7.61, SE 3.46, t_225_=2.20, 95% CI 0.79-14.43, *P*=.03). Age and the other education categories were not significant predictors (age, *P*=.19 and education, *P*=.40).

The significance of the regression coefficient for the BrainHQ composite indicates that, for every 1-unit increase in the BrainHQ composite score, AFQT scores increased by approximately 15.40 points. The intercept was also significant (β=51.26, SE 9.67, t_225_=5.30, 95% CI 32.20-70.32, *P*=.001), representing the estimated AFQT score when the BrainHQ composite *z* score was 0 (51.26). This relationship can be expressed as follows:

AFQT = (BrainHQ composite *z* score) × 15.40 + 51.26

No significant differences were observed between included and excluded participants on the AFQT or demographic variables (age, *P*=.43; sex, *P*=.74; ethnicity, *P*=.35; and education, *P*=.67), suggesting that missing data were unlikely to bias the regression estimates.

All preplanned partial correlations between the BrainHQ and Penn CNB subtests, controlling for age and education, were positive and of small-to-moderate magnitude: Mind Bender and CPT (*r*=0.17, 95% CI 0.03-0.31, *P*=.02), Face to Face and MEDF36 (*r*=0.25, 95% CI 0.12-0.37, *P*<.001), Pattern Detective and PVRT (*r*=0.20, 95% CI 0.06-0.33, *P*=.004), and Auditory Paired Associates and CPW (*r*=0.17, 95% CI 0.03-0.30, *P*=.01).

All correlations were statistically significant using an uncorrected α=.05, as defined in the analysis plan (W Schlinsog et al, unpublished data, 2026). The prespecified hypothesized construct overlap limited the inflation of the familywise error rate; therefore, no formal correction was applied. However, under a conservative Bonferroni-corrected familywise error rate of 0.01, only the Face to Face and MEDF (*P*<.001) and Pattern Detective and PVRT (*P*=.004) associations would remain statistically significant.

**Table 1:** Baseline characteristics of National Guard recruits participating in the ONR^a^ Neuropsychometrics study (N=267).

Characteristic			Values
Age (years), mean (SD)			19.13 (3.27)
Education, n (%)			
	Some high school	93 (34.8)	
	GED^b^/high school diploma	88 (33.0)	
	Some college or associate degree	60 (22.5)	
	4-Year college degree or higher	16 (6.0)	
	Declined	10 (3.7)	
Gender, n (%)			
	Male	178 (66.7)	
	Female	86 (32.2)	
	Declined	3 (1.1)	
Race, n (%)			
	White	164 (61.4)	
	Black or African American	22 (8.2)	
	American Indian or Alaska Native	0 (0.0)	
	Asian	40 (15.0)	
	Other	8 (3.0)	
	Native Hawaiian or Other Pacific Islander	0 (0.0)	
	Multiracial	28 (10.5)	
	Declined	5 (1.9)	
Ethnicity, n (%)			
	Not of Hispanic Origin	230 (86.1)	
	Hispanic	34 (12.7)	
	Declined	3 (1.1)	
AFQT^c^, percentile score (SD; range)			70.45 (19.54; 20-99)
Penn CNB^d^, cognitive domain, subtest name, mean score (SD; range)			
	Executive Function, CPT^e^, d′	2.83 (0.98; –0.10 to 4.72)	
	Word Memory, CPW^f^, d′	2.05 (0.90; –1.43 to 3.96)	
	Social Cognition, MEDF36^g^, total correct responses	26.80 (3.52; 16 to 34)	
	Reasoning, PVRT^h^, total correct responses	3.89 (1.64; 0 to 8)	
BrainHQ, cognitive domain, subtest name, mean score (SD; range)			
	Executive Function, Mind Bender (ms)	1248.22 (1852.96; 151 to 9410)	
	Word Memory, Auditory Paired Associates (set size)	4.25 (2.42; 1.5 to 12)	
	Social Cognition, Face to Face (ms)	2651.25 (1756.43; 89 to 5623)	
	Reasoning, Pattern Detective (difficulty)	2.68 (1.84; 1 to 8)	
	Working Memory, Target Tracker (set size)	3.43 (1.14; 1 to 6.5)	
	Visual Speed, Double Decision (ms)	693.98 (786.48; 32 to 3162)	
	Auditory Speed, Sound Sweeps (ms)	209.14 (310.44; 16 to 1000)	

^a^ONR: Office of Naval Research.

^b^GED: General Educational Development.

^c^AFQT: Armed Forces Qualification Test.

^d^Penn CNB: Penn Computerized Neurocognitive Battery.

^e^CPT: Penn Continuous Performance Test.

^f^CPW: Penn Word Memory Test.

^g^MEDF36: Penn Measured Emotion Differentiation Test (36 items).

^h^PVRT: Penn Verbal Reasoning Test.

**Figure 3:**
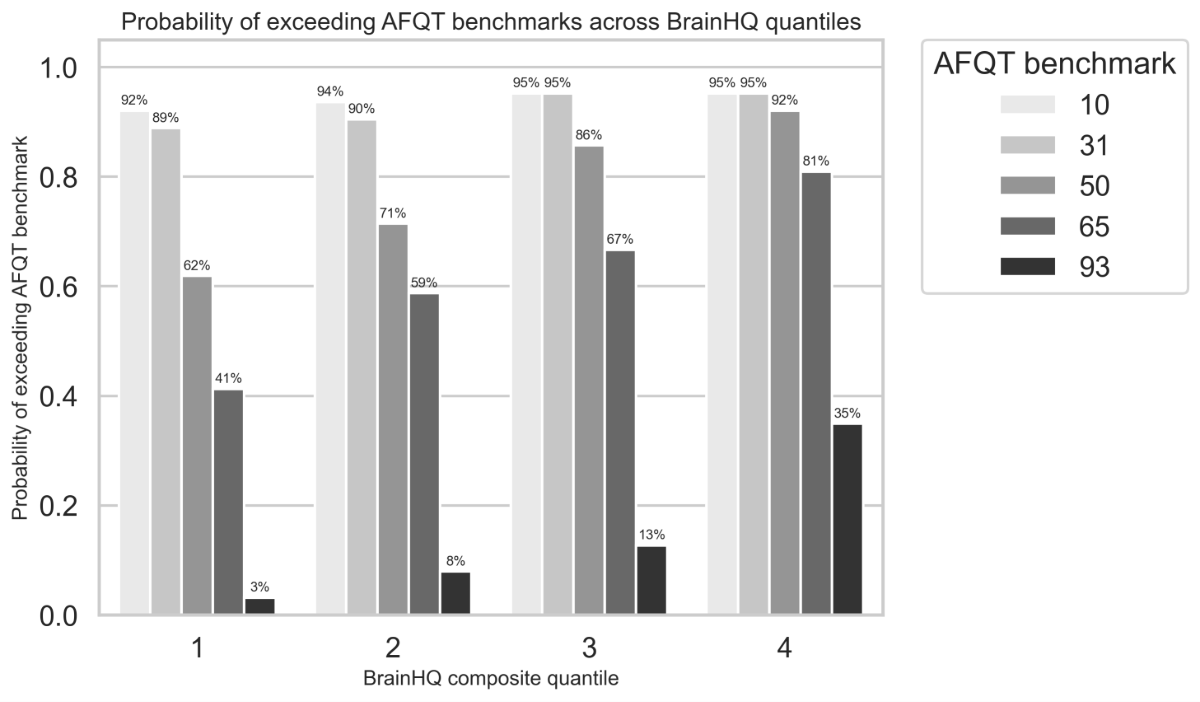
Proportion of participants scoring at or above established Armed Forces Qualification Test (AFQT) eligibility bands within each BrainHQ composite quartile. Higher BrainHQ performance was associated with a graded increase in the likelihood of attaining higher operationally relevant AFQT benchmarks.

The resulting probabilities from the BrainHQ composite and AFQT benchmarks are reported in Table 2 and visualized in Figure 3. In the lowest BrainHQ quartile, 58 out of 63 (92%) participants obtained an AFQT score of ≥10, 56 (89%) obtained ≥31, 39 (62%) obtained ≥50, 26 (41%) obtained ≥65, and only 2 (3%) obtained ≥93. By contrast, in the highest quartile, 60 out of 63 (95%) participants obtained an AFQT score of ≥10, 60 (95%) obtained ≥31, 58 (92%) obtained ≥50, 51 (81%) obtained ≥65, and 22 (35%) obtained ≥93. Intermediate quartiles showed a graded increase in probabilities across AFQT benchmarks. Framed differently, participants in the lowest BrainHQ quartile exceeded an AFQT cutoff score of 50 approximately 62% (39/63) of the time, whereas those in the highest quartile exceeded the same threshold 92% (58/63) of the time. Similarly, the highest-performing quartile had a 35% (22/63) probability of meeting the most stringent AFQT benchmark (≥93), compared with only 3% (2/63) for the lowest quartile.

**Table 2:** Percentage of participants who scored at or above established AFQT^a^ eligibility bands within each BrainHQ composite quartile, ranging from the lowest-performing quartile (1) to the highest-performing quartile (4).

BrainHQ composite quartile and AFQT benchmark^b^, %		Values^c^
1		
	10	58/63 (92)
	31	56/63 (89)
	50	39/63 (62)
	65	26/63 (41)
	93	2/63 (3)
2		
	10	59/63 (94)
	31	57/63 (90)
	50	45/63 (71)
	65	37/63 (59)
	93	5/63 (8)
3		
	10	60/63 (95)
	31	60/63 (95)
	50	54/63 (86)
	65	42/63 (67)
	93	8/63 (13)
4		
	10	60/63 (95)
	31	60/63 (95)
	50	58/63 (92)
	65	51/63 (81)
	93	22/63 (35)

^a^AFQT: Armed Forces Qualification Test.

^b^AFQT benchmarks were defined as ≥10 (representing individuals typically ineligible for enlistment), ≥31 (eligible with some restrictions), ≥50 (general eligibility across roles), ≥65 (high eligibility across most roles), and ≥93 (qualifying for any military occupational specialty).

^c^Presented as n above benchmark/N (% probability exceeding benchmark).

## Discussion

### Principal Findings

The BrainHQ brain health battery is the first brief, self-administered, and highly scalable cognitive assessment to demonstrate preliminary construct validity with measures of military vocational aptitude and neurocognitive domains associated with cognitive resilience. In a large observational study of National Guard soldiers, higher performance on the BrainHQ composite measure was positively associated with eligibility and aptitude as indexed by the AFQT (Cohen *f*^2^=0.25, medium effect size) after controlling for age and education. Although the AFQT is influenced by many factors (eg, education, socioeconomic status), a significant proportion of its variance was accounted for by a single computerized brain health composite measure, demonstrating a moderate effect size and supporting its potential practical utility as a proxy for vocational placement. Higher BrainHQ composite performance further corresponded to progressively greater probabilities of meeting operationally relevant AFQT aptitude benchmarks. Preliminary construct validity for resilience was supported by small-to-moderate effect sizes (Cohen *q*=0.17-0.26) between BrainHQ and the Penn CNB despite an 18-24-month gap between administrations, suggesting modest but meaningful convergence across conceptually related constructs. These effect sizes are consistent with those reported in prior assessment validation studies using meaningful biological and cognitive performance outcomes [28,36]. Overall, the findings provide initial evidence that the battery may serve as a behavioral index of military career aptitude and cognitive factors associated with resilience.

In the context of cognitive testing, transfer refers to the degree to which performance on 1 task is associated with performance on a distinct task. The 4 BrainHQ brain health subtests examined in this study were designed with experimental paradigms, stimulus types, task demands, and scoring metrics that differed from those used in the 4 Penn CNB subtests. The Penn CPT used a go/no-go paradigm with alphanumeric stimuli and was scored using a sensitivity index, whereas BrainHQ’s Mind Bender used a task-switching paradigm with pictorial stimuli and exposure-duration thresholds. Similarly, the Penn CPW involved list learning and delayed recall of visually presented words, again using sensitivity for scoring, whereas BrainHQ’s Auditory Paired Associates assessed associative memory for spoken word pairs presented at speed, with performance defined as the number of pairs recalled. The Penn MEDF36 used a face-morphing paradigm requiring participants to discriminate gradations of emotional intensity, measured as total correct responses, whereas BrainHQ’s Face to Face assessed emotion identification across different individuals expressing the same emotion, with performance measured using exposure-duration thresholds. Finally, the Penn PVRT used a verbal analogy paradigm scored by total correct responses, whereas BrainHQ’s Pattern Detective used a visual matrix reasoning paradigm involving abstract patterns presented at speed, with scoring based on pattern complexity. Differences in implementation parameters and task delivery across auditory and visual modalities likely attenuated the strength of the observed associations. Nevertheless, performance on domain-similar BrainHQ and Penn CNB measures was significantly associated. This preliminary convergence supports BrainHQ’s information-processing approach as capturing cognitive processes that overlap with those measured by more traditional neuropsychological instruments.

### Significance and Implementation

The BrainHQ brain health assessment uses a standardized, automated format that allows for consistent administration and interpretation across diverse military settings. This design makes it well suited for large-scale cognitive screening and for identifying strengths and vulnerabilities early in a service member’s career. Unlike the ASVAB/AFQT, which emphasizes declarative and academic knowledge and has been validated as a predictor of military training and occupational outcomes, BrainHQ assessments are designed to measure how efficiently the brain processes incoming information under time pressure. All assessments rely on rapidly presented visual and auditory stimuli, sometimes as brief as 16 ms, which limits the use of strategies such as writing down correct responses for later entry. This approach may complement existing military evaluation systems.

Variations of the BrainHQ assessment, including components of the current battery, have been previously deployed in diverse military contexts. These applications include work with the US Special Operations Command to characterize cognitive strengths and weaknesses in operators, longitudinal monitoring of the cognitive effects of wearable technologies with the US Army, qualification and ongoing training for drone operators with the Canadian Explosives Technicians Association, and screening of recruits in the Italian Army. Collectively, these initiatives highlight the adaptability of the platform across different military roles and environments, ranging from highly specialized cohorts to large-scale recruitment settings requiring the efficient evaluation of large numbers of individuals. This study demonstrates significant associations between BrainHQ performance and AFQT scores, thereby extending prior applications of cognitive assessment to the foundational stage of enlistment. The observed preliminary construct validity with standardized neurocognitive measures further supports BrainHQ’s design principles as a scientifically valid approach for evaluating neuropsychologically defined cognitive domains. These findings, combined with the success of ongoing field deployments, support the feasibility of BrainHQ assessments across multiple stages of the military pipeline, from recruitment and training to longitudinal monitoring.

### Limitations

First, participants in this study were recruited from ARMOR, which drew participants from a defined geographic area. This recruitment strategy, while operationally necessary, may have limited the applicability of the findings to military personnel from other states or branches of service. Second, variability in testing environments and hardware configurations may have introduced uncontrolled sources of measurement error. Differences in device type, display size, and input method could influence exposure duration and response precision. Likewise, environmental factors such as background noise, ambient distractions, and network connectivity may affect engagement and performance. Although these factors are inherent to unsupervised digital assessments, future work should aim to quantify their effects and demonstrate that their influence on performance is minimal. Third, participant identity was not independently verified, and it is possible that an individual other than the enrolled service member completed the BrainHQ assessments, introducing a source of potential measurement error that should be considered when interpreting the findings. There were, however, no performance-based incentives, compensation, or recognition associated with test scores that would be expected to motivate substitution or intentional misrepresentation. Fourth, the study experienced a 22.4% (77/344) attrition rate between consent to the ONR Neuropsychometrics study and self-initiation of related study activities, which may have introduced selection bias. Fifth, because the BrainHQ, AFQT, and Penn CNB measures were collected through separate studies, they were not administered concurrently, which likely attenuated the observed effects. The temporal separation of AFQT at enlistment, the Penn CNB shortly thereafter, and BrainHQ approximately 18-24 months after BCT suggests that these findings reflect associations among underlying cognitive constructs rather than concurrent validity. However, the emergence of significant relationships despite substantial collection lags suggests some degree of stability in cognitive performance across time and contexts.

### Strengths

One of the key strengths of this study is the use of a brief, self-administered battery designed to evaluate capabilities relevant to military success, such as vocational aptitude. The protocol is standardized and scalable, allowing deployment across both laboratory and field settings.

### Future Directions

Future studies with temporally aligned or prospective data collection will be necessary to disentangle construct-level associations from the temporal stability of performance and to more rigorously evaluate predictive and concurrent validity. These investigations should examine the predictive validity of performance on the BrainHQ assessment in relation to other measures of resilience and mission-critical outcomes, including successful completion of BCT, advancement through military career pathways, and sustained cognitive performance under operational demands. The generalizability of cognitive performance metrics across demographic subgroups should also be evaluated to determine whether the predictive relationships between cognitive assessment scores and operational outcomes are consistent across sex, race and ethnicity, and educational attainment. Such analyses would help identify potential subgroup differences or sources of differential prediction that could introduce unintended bias. Longitudinal applications may further support proactive brain health monitoring throughout military service to inform individualized interventions and force-wide aptitude and resilience strategies.

### Conclusions

The BrainHQ brain health assessment battery demonstrates preliminary construct validity with an established measure of vocational aptitude and with neurocognitive domains associated with cognitive resilience in military research contexts. This tool can be completed by any applicant or service member using an internet-connected device, allowing widespread implementation at minimal logistical and financial cost.

## Abbreviations

AFQT: Armed Forces Qualification Test
ARMOR: Advancing Research on Mechanisms of Resilience
ASVAB: Armed Services Vocational Aptitude Battery
BCT: Basic Combat Training
CPT: Penn Continuous Performance Test
CPW: Penn Word Memory Test
GED: General Educational Development
HIPAA: Health Insurance Portability and Accountability Act
LOE5: Line of Effort 5
MEDF36: Penn Measured Emotion Differentiation Test (36 items)
NIH: National Institutes of Health
ONR: Office of Naval Research
Penn CNB: Penn Computerized Neurocognitive Battery
PVRT: Penn Verbal Reasoning Test
UMN: University of Minnesota

## Data Availability

The data are not publicly available because of security restrictions. Datasets are available from MP upon reasonable request and subject to approval by the University of Minnesota or the US Department of Veterans Affairs. Analysis scripts are available from MA upon reasonable request.
